# Surgical Treatment of Central Giant Cells Lesions in the Maxilla: Case Report

**DOI:** 10.1016/S1808-8694(15)30956-3

**Published:** 2015-10-19

**Authors:** Dmitry José de Santana Sarmento, Jalber Almeida dos Santos, Lúcia Helena Marques de Almeida Lima, Marcelino Guedes de Lima, Gustavo Pina Godoy

**Affiliations:** 1 Master's degree student of oral pathology, UFRN; 2 Master's degree student of dentistry, UEPB; 3 Full professor, UEPB; 4 Full professor, UEPB; 5 UFRN. Full professor of oral pathology, UEPB

**Keywords:** granuloma, giant cell, pathology

## INTRODUCTION

The central giant cell granuloma (CGCG) consists of a benign non-neoplastic proliferative lesion comprising about 7% of jaw lesions. It affects mostly children and young adults; the female to male ratio is 2:^1^.^1^, ^2^, ^3^

The CGCG is generally asymptomatic; its clinical behavior may be non-aggressive or aggressive. This lesion may invade the floor of the maxillary sinus, the orbit, and the nasal fossae. There may also be facial asymmetry, nasal deviation, and mobility of associated teeth.^1,^^4^

The etiopathogeny is unclear; there are different clinical presentations and several forms of treatment.^2^ The radiographic aspect is a well-defined uni- or multilocular radiolucent lesion, which may expand to the cortical plate and cause dislocation of theeth.^3^

The diagnostic hypotheses depend on the aggressiveness, development rate, inflammation, pain, mobility of teeth and other manifestations of the lesion. The differential diagnosis includes peripheral giant cell granulomas, aneurismatic bone cysts, and the brown tumor of hyperparathyroidism.^4^

The histology of CGCG of the jaws shows multinucleated giant cells and round, oval or spindle-shaped mononucleated cells within fibrous connective tissue, associated with focal hemorrhage and significant vascularization.^2^

Treatments are: excision biopsies, curettage with safety margins, and partial or full resection of the affected bone; injecting corticosteroids into the affected region has been successful.^1,^^5^

Supportive care includes antibiotics, analgesics, and corticosteroids for about 10 days after surgery; postoperative monitoring with radiographic exams is needed to assess disease progression.^1^

Based on these ideas, the purpose of this study was to report a case of CGCG, with an emphasis on the clinical procedures, radiography and laboratory tests for the diagnosis and surgical treatment.

## CASE REPORT

A female white patient aged 33 years presented at the buccomaxillofacial surgery and trauma unit of the Pedro I Hospital in the city of Campina Grande, PB, complaining of loss of stability of her dental prosthesis and increased volume on the left side of the jaw. The patient thought that this was due to the recent removal of a tooth, which the patient described as rather traumatic.

No changes were seen in the extra-oral physical examination. Within the mouth, the left maxillary region was enlarged, with expanded bone cortical plates. Upon palpation, the lesion was hard and showed intra-osseous extension.

**Figure 1:**
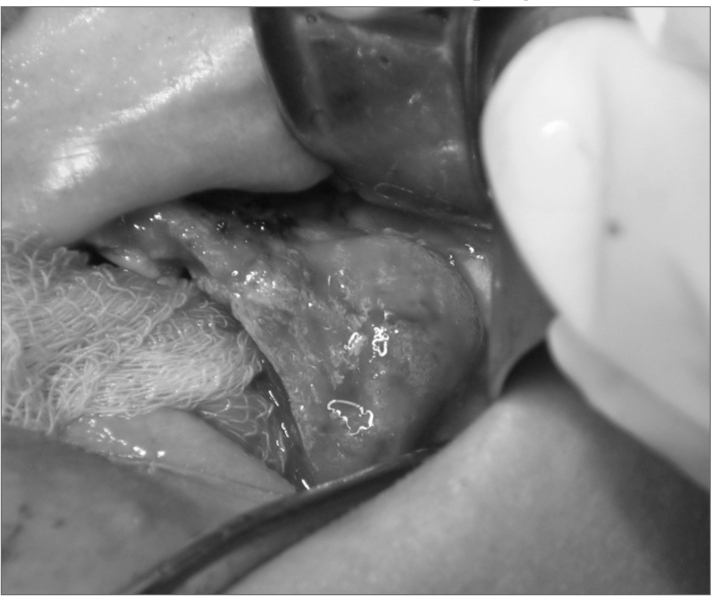
Surgical treatment of a maxillary central giant cell granuloma: case report - findings at surgery.

The panoramic radiograph and computed tomography revealed a radiopaque mass containing radiolucent images. The lesion was removed surgically with a safety margin, and meticulous curettage was carried out to improve the prognosis. The patient was admitted into hospital for the procedure (Fig. 1); before surgery, an incision biopsy had been done in an outpatient setting. The specimen was sent to the histopathology department, which issued a diagnosis of CGCG. Medical therapy consisted of amoxicillin 500mg (antibiotic) - one capsule PO every 8 hours, dipirone 500mg (analgesic) - one tablet PO every 6 hours, and dexamethasone 0.75ml (corticosteroid) - one table PO every 12 hours.

Histological sections revealed connective tissue fragments with two cell populations (mononucleated and multinucleated). Mononucleated cells were spindle-shaped, with elongated nuclei and clear cytoplasm. The multinucleated giant cells contained several nuclei and a large eosinophilic cytoplasm.

The patient was monitored during one month, after which routine visits during 6 months were scheduled; the postoperative period was uneventful.

## DISCUSSION

Ahmed et al.^2^ and Ciorba et al.^6^ have stated that CGCG is more common in females aged below 30 years, as supported by the present case. Although the lower jaw is the main site for CGCG, 1,3 the patient presented a lesion in the maxilla, as also highlighted by Uzbek and Mushtaq.^5^ Güngörmüs and Akgül^3^ stated that 22% of CGCG patients were fully edentulous, which was also the case of our patient.

The etiology of CGCG remains unclear; some factors, such as trauma and multiple exodontia, may be associated with this disease;^1,^^5^ these findings were present in the history of the present case.

The patient was asymptomatic, there was no bleeding, the cortical plates were bulging, there was mild facial asymmetry, and the lesion was palpable in the examination of the mouth; these findings were also reported by other authors such as Neville et al.^4^ and Ciorba et al.^6^

The histopathology of CGCG generally is described as populations of mononucleated and multinucleated cells; inflammation may also be found.^2^, ^3^, ^4^ These findings were present in the study specimen.

Surgery is the traditional and generally accepted approach for the treatment of CGCG.^5,^^6^ It was the method of choice in the present case; the lesion was removed, followed by meticulous curettage to reduce the risk of recurrences. This approach was also used by Tasar et al.^1^ Uzbek and Mushtaq.^5^ Corticosteroids, antibiotics and analgesics were also given to relieve postoperative symptoms and improve the prognosis.

## CONCLUSION

The CGCG is an infrequent disease of unclear etiology. Although benign, it may be high destructive. The conclusive diagnosis is made by histopathological studies. The treatment of choice should be selected according to the peculiarities of each case.
